# Transcriptional adaptation of staphylococci during colonization of the authentic human environment: An overview of transcriptomic changes and their relationship to physiological conditions

**DOI:** 10.3389/fcimb.2022.1062329

**Published:** 2022-11-17

**Authors:** Marc Burian, Christiane Wolz, Amir S. Yazdi

**Affiliations:** ^1^ Department of Dermatology and Allergology, RWTH University Hospital Aachen, Aachen, Germany; ^2^ Interfaculty Institute of Microbiology and Infection Medicine, University of Tübingen, Tübingen, Germany; ^3^ Cluster of Excellence EXC 2124 “Controlling Microbes to Fight Infections”, University of Tübingen, Tübingen, Germany

**Keywords:** bacterial adaptation, gene expression, *in vivo*, global regulators, nasal colonization, skin colonization, virulence factors, host pathogen interaction

## Abstract

Staphylococci are commensals of human skin and mucous membranes, but some species can also cause serious infections. Host niches during both colonization and infection differ greatly and are characterized by specific environmental conditions (pH, temperature, oxygen, nutrient availability, and microbiota) that can affect gene expression and virulence of microbes. To successfully occupy extremely different habitats at different anatomical sites, Staphylococci are equipped with a variety of regulatory elements that allow specific adaptation to the changing environments. Not surprisingly, gene expression *in vivo* can be significantly different from the expression pattern observed *in vitro*. Niche specific stimuli that influence the bacterial ability to either cause infection or maintain colonization are only partially understood. Here, we describe habitat specific conditions and discuss the available literature analyzing staphylococcal gene expression, focusing on *Staphylococcus aureus* and *S. epidermidis* during colonization of the nose and skin.

## Introduction

*Staphylococci* are commensals of human skin and mucous membranes but can also cause serious infections (Gordon and Lowy, 2008). The versatility to colonize and infect various human body sites is facilitated by a complex transcriptional regulatory network. Regulation is achieved by > 100 regulatory elements, including two component systems (TCSs), alternative sigma factors, transcription factors and small regulatory RNAs (sRNAs) (Bleul et al., 2021). Recent advances in transcriptomics and molecular analyses revealed a close link between metabolic adaptation and virulence gene expression (Prince and Wong Fok Lung, 2020; Rudra and Boyd, 2020). For *Staphylococcus aureus* co-regulated genes were grouped into 29 independently modulated sets of genes (i-modulons) (Poudel et al., 2020), and for many regulators, prototypic target genes are well defined based on known binding motifs (Novichkov et al., 2013). However, which signals are perceived and how they are transmitted is often less clear (Bleul et al., 2021).

To obtain a better understanding of the adaptive processes *in vivo*, several approaches were chosen. First, the *in vivo* conditions can be defined e.g. through metabolomics and the information used to establish adapted growth media to mimic *in vivo* conditions (Krismer et al., 2014). Second, organoids or the use of explants are useful tools to unravel host-bacterial interactions (Burian et al., 2021; Cruz et al., 2021). Third, analyses of gene expression in *ex vivo* samples can decipher which regulatory circuits are active and allow conclusions about the growth conditions encountered *in vivo* (Burian et al., 2010b; Chaves-Moreno et al., 2016). The major limitation of such analyses in the authentic human environment is the difficulty in obtaining enough RNA at high purity and data normalization. Here, we summarize the current knowledge of conditions prevailing in two important staphylococcal habitats - the nose and the skin - and describe how *in vivo* gene expression may be determined by these conditions.

## Nasal colonization

### The nose environment

*Vestibulum nasi* forms the main ecological niche for *S. aureus* (Wertheim et al., 2005). According to the traditional view, the nasal epithelium consists of basal, secretory and ciliated cells. However, single-cell RNA sequencing revealed more than 10 different cell types, some of which were highly specialized. Club and goblet cells form nasal secretions (Hewitt and Lloyd, 2021), which are mainly composed of water (95%), mucin glycoproteins (2%), salt (1%), lipids (1%) and various proteins (1%) (Kaliner et al., 1984). Nasal secretions, along with the nasal microbiome, contribute to the first layer of host defense. Mucin glycoproteins provide binding sites for interactions with microbial structures and thus contribute to the sequestration of pathogens (Fahy and Dickey, 2010). In nasal secretions, in addition to the numerous antimicrobial proteins, of which lysozyme, lactoferrin, and the secretory leukoprotease inhibitor are the most abundant, immunoglobulins (IgA, IgE and IgG), and α- and β-defensins are also present (Cole et al., 1999; Tomazic et al., 2020). However, the detectability of defensins is highly donor dependent (Cole et al., 1999; Preiano et al., 2018).

Krismer and colleagues determined the metabolites in nasal secretions (Krismer et al., 2014) and found that nutrients were present in rather low amounts compared to the amounts in plasma (Nasset et al., 1979) and sputum from cystic fibrosis patients (Palmer et al., 2007). Of the carbohydrates in nasal secretions, glucose is the major monosaccharide (35 µM - 1 mM), and urea is the most abundant organic substance (2.5 - 7.5 mM). Interestingly, while most amino acids in nasal secretions are present at an average concentration of 50 - 150 µM, methionine, glutamine, tyrosine, isoleucine, asparagine and aspartate were nondetectable. In addition, no lactate and only trace amounts of fatty acids were detected (Krismer et al., 2014). The levels of essential metals, such as iron, zinc, and manganese, are also low (Krismer et al., 2014). Sodium chloride was present in nasal secretions at physiological concentrations (~150 mM) (Vanthanouvong and Roomans, 2004), and the mean nasal pH was 6.5 (± 0.5) (Kim et al., 2013). Based on these data, a synthetic nasal medium (SNM) was composed and gene expression in SNM versus BM complex medium compared (GSE43712) (Krismer et al., 2014). Key genes were expressed in SNM in a similar way as in the human nose, indicating that SNM represents a suitable surrogate environment for *in vitro* simulation studies.

### Bacterial adaptation to the nose environment

*S. aureus* and coagulase-negative staphylococci (CoNS), such as *S. epidermidis*, are core members of the nasal microbiome (Liu et al., 2015) and thus have evolved to cope with that specific environment. In contrast to *S. epidermidis*, only approximately 20% of the healthy human population is persistently colonized with *S. aureus* in the nose (Van Belkum et al., 2009). Whereas *S. aureus* usually has only one strain colonizing the host (Vandenbergh et al., 1999; Van Belkum et al., 2009), recent metagenomics studies for *S. epidermidis* show a large heterogeneity at the strain level within a host niche (Both et al., 2021; Severn and Horswill, 2022). Nevertheless, virulence regulators such as the *agr* quorum sensing system are conserved among staphylococci (Thoendel et al., 2011). Individual virulence factors such as the sphingomyelinase gene of *S. epidermidis* are also highly conserved, as demonstrated in a large cohort of skin isolates from healthy volunteers (Zheng et al., 2022).

*Ex vivo* gene expression analyses are promising approaches to gain insight into niche adaptation. There are a few studies describing gene expression during nasal colonization of *S. aureus* (Burian et al., 2010b; Burian et al., 2012; Song et al., 2012; Krismer et al., 2014; Chaves-Moreno et al., 2016) or *S. epidermidis* (Teichmann et al., 2022). In most studies, transcript analyses were performed directly on nasal swabs from persistently colonized individuals. Gene expression was measured by qRT-PCR and compared to the expression pattern of the isogenic strain(s) grown *in vitro*. Similar *in vivo* transcriptional profiles were observed for most of the genes analyzed when specimens from different volunteers or follow-up specimens from the same volunteer were compared (Burian et al., 2010b). A similar *in vivo* expression pattern was also detected in a cotton rat model (Burian et al., 2010a) and in a human airway epithelial coculture model (Kiedrowski et al., 2016).

In one study, meta-transcriptomics was applied to obtain a more comprehensive overview of the *in vivo* gene expression of *S. aureus* (GSE73485) (Chaves-Moreno et al., 2016). Reads were compared to data obtained for two non-isogenic reference strains (USA300 LAC or IPL32) grown *in vitro*. Cluster analysis revealed that all *in vivo* transcriptomes differed substantially from those of the *in vitro*-grown *S. aureus* strains. However, large differences were obvious between the five *in vivo* transcriptomes, with them sharing only >55% similarity (Chaves-Moreno et al., 2016). Based on the known large strain differences between *S. aureus* isolates (Lindsay, 2010; Lindsay, 2014), it is not surprising that the *in vivo* transcription differs from transcription of laboratory strains. Thus, comparison with the isogenic strains grown *in vitro* (Burian et al., 2010b) is more informative about habitat specific changes in gene expression.

**Figure 1:**
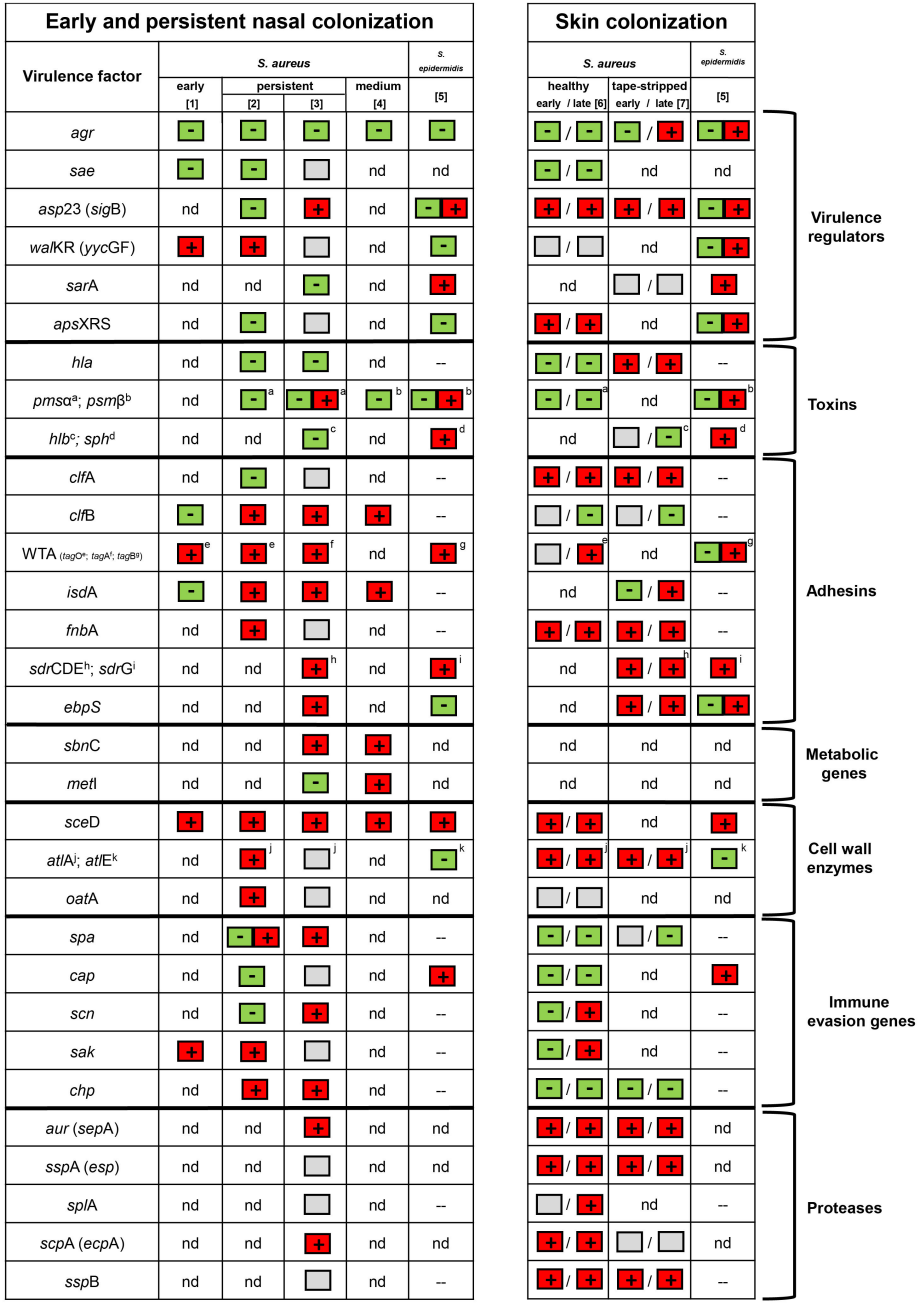
Transcriptional patterns in the nose and skin of *S. aureus* and *S. epidermidis* based on the following literature: 1 = (Burian et al., 2010a); 2 = (Burian et al., 2010b); 3 = (Chaves-Moreno et al., 2016); 4 = (Krismer et al., 2014); 5 = (Teichmann et al., 2022); 6 = (Burian et al., 2021); and 7 = (Cruz et al., 2021). Genes upregulated were marked with a + (box with red background), while genes downregulated were marked with a – (box with green background). The box with a gray background indicates no regulation. A box with + and – (red and green background) indicates heterogeneous transcription. nd = not determined. – = not present.

Nevertheless, comparison of the analyses performed thus far revealed some common themes (**Figure 1**). Nasal colonization of *S. aureus* is clearly linked to increased expression of adhesin genes (*clf*B, *fnb*A, *sdr*CDE, *isd*A, *sas*F, *ebp*S, *atl*A, and *eap*) and wall teichoic acid (WTA) biosynthesis genes (measured by *tag*O (Burian et al., 2010b) and by *tag*A (Chaves-Moreno et al., 2016)). For *S. epidermidis*, genes encoding the fibrinogen binding protein SdrG and WTA (measured by *tag*B) were upregulated (Teichmann et al., 2022). Some of the genes that encode host defense subversion, such as staphylokinase (*sak*), chemotaxis inhibitory protein (*chp*) and protein A (*spa*), were also expressed *in vivo* (Burian et al., 2010b; Chaves-Moreno et al., 2016). Expression of the *S. aureus cap* operon (encoding enzymes for capsular polysaccharide synthesis) was variable between and within specimens (Burian et al., 2010b; George et al., 2015). The *capBCAD* operon of *S. epidermidis* is responsible for the production of poly-γ-glutamic acid (γ-PGA) and highly expressed during colonization (Teichmann et al., 2022). Since γ-PGA is also present in other CoNS (Kocianova et al., 2005; Watanabe et al., 2018), this, together with the observed high transcription in *S. epidermidis*, suggests a species-wide protective mechansims for CoNS.

It can be assumed that bacteria encounter specific stress conditions in the nasal environment. There is a clear indication that *S. aureus* is iron-restricted *in vivo*. Iron-regulated genes, such as *isd*A, were found to be highly expressed in all studies (Burian et al., 2010b; Krismer et al., 2014; Chaves-Moreno et al., 2016) (**Figure 1**). Further indications of iron restriction are the high transcription of genes encoding enzymes for siderophore synthesis and their respective transport systems (*sir* and *hts*) (Chaves-Moreno et al., 2016).

The induction of genes protecting against reactive oxygen species (*kat*A, *ahp*C) and genes forming the compatible solute glycine betaine (*bet*A, *bet*B) indicate that *S. aureus* is exposed to oxidative and osmotic stress, respectively (Chaves-Moreno et al., 2016).

The metabolic state of *in vivo*-grown bacteria is still not well understood. For *S. epidermidis*, sphingomyelinase (*sph*) activity provides nutrients to the bacterium by cleaving sphingomyelin into phosphocholine and ceramide (Zheng et al., 2022). This was supported by the unusual high *sph* expression in *S. epidermidis* nose and skin specimens (Teichmann et al., 2022). Interestingly, the expression of genes encoding tricarboxylic acid cycle enzymes of *S. epidermidis* was low *in vivo*. Since these enzymes are usually suppressed under nutrient-rich conditions (Somerville and Proctor, 2009), the results indicate good nutrient supply for *S. epidermidis* in its natural habitat (Teichmann et al., 2022). This seems to contrast with the observation that in SNM medium, the growth of *S. epidermidis* is inferior to that of *S. aureus* (Krismer et al., 2014). One can speculate that the high activity of sphingomyelinase contributes to the growth advantage of *S. aureus in vivo* since the substrate, sphingomyelin, is missing in SNM.

The host nasal environment activates specific metabolic pathways required for long-term colonization. For example *de novo* synthesis of methionine and significant upregulation of several amino acid biosynthesis genes was observed during *S. aureus* nose colonization (Krismer et al., 2014). A shift toward lipid and amino acid metabolism was also detected in an airway epithelial coculture model (Kiedrowski et al., 2016).

The activity of pleiotropic regulators should be informative to obtain further insights into the environmental conditions encountered *in vivo*. Major regulatory systems driving the expression of virulence genes, such as the *agr* quorum-sensing system or the virulence gene regulatory system *sae*PQRS, were found to be inactive during nasal colonization (Burian et al., 2010a; Burian et al., 2010b; Pynnonen et al., 2011; Song et al., 2012; Teichmann et al., 2022). The inactivity of the SaePQRS system might be due to the low abundance of α-defensins, which were shown to be important ligands for the activation of the histidine kinase SaeS (Geiger et al., 2008). The Agr system might be inactive due to the low bacterial density, the inhibition by interfering *staphylococcal* species (Jenul and Horswill, 2019) or the presence of hemoglobin (Pynnonen et al., 2011). The inactivity of both virulence regulators indicates that *S. aureus* is kept in a nontoxic state during colonization.

The essential two-component system WalKR seems to be active during colonization (Burian et al., 2010a; Burian et al., 2010b). To date, the signal for WalKR activation is still not well defined but probably involves some disturbance of cell-wall metabolism (Bleul et al., 2021). The defined WalKR target gene *sce*D coding for a lytic transglycosylase (Dubrac et al., 2007) is the most prominent and reproducible *in vivo* activated gene in *S. aureus* (Burian et al., 2010a; Burian et al., 2010b; Krismer et al., 2014; Chaves-Moreno et al., 2016; Kiedrowski et al., 2016) and *S. epidermidis* (Teichmann et al., 2022). Given the clear involvement of WalKR and especially its target gene *sce*D (**Figure 1**), this could be a useful target to prevent colonization/infection. Therefore, further research is needed to decipher the exact role of *sce*D and its regulatory system WalKR. For *S. epidermidis* the accessory staphylococcal regulator A (*sar*A) also seems to play an important role during colonization (Teichmann et al., 2022).

Small RNAs are involved in the posttranscriptional regulation of metabolic pathways and in responses to stress and virulence (Menard et al., 2021). The expression levels of five sRNAs of *S. aureus* were quantified during human colonization and infection. The expression level of the Agr effector molecule RNAIII was again much lower *in vivo*, supporting that the system is largely kept inactive (Song et al., 2012).

One important question is whether the bacteria divide actively and at what rate of growth. Evidence, such as the high expression of cell envelope components (*tag*O, *tar*K, *atl*A, *sce*D and *oat*A)indicates that *S. aureus* is not in a dormant state. Moreover, genes expressed during the exponential growth phase *in vitro* are highly expressed in the human nose (Burian et al., 2010b). The expression levels of the sRNAs *in vivo* also resembled those obtained at the exponential phase or late exponential phase of growth *in vitro* (Song et al., 2012). The assumption that *S. aureus* is rapidly dividing during colonization is also supported by the distribution of sequencing coverage along the staphylococcal chromosome and the rate of mutational accumulation (Szafranska et al., 2019). This indicates that colonization of the human upper respiratory tract is characterized by a highly dynamic equilibrium between bacterial growth and removal.

## Skin colonization

### The skin environment

Human skin represents a highly variable organ that varies in temperature, pH, moisture and sebum content, creating different niches for microorganisms (Grice and Segre, 2011). The outermost layer, the stratum corneum, consists of the upper layers of corneocytes and is rich in ceramides, cholesterol, and free fatty acids. The hydrophobic and viscous sebum produced by sebaceous glands located in the dermis consists of a mixture of nonpolar lipids, such as triglycerides, wax esters, squalene, fatty acids and smaller amounts of cholesterol and diglycerides (Pappas, 2009).

The microenvironment on the skin is also influenced by sweat produced by eccrine and apocrine glands. Eccrine glands excrete ions and various proteins and peptides, some of which are also involved in innate host defense mechanisms, such as DNase I, lysozyme, and dermcidin (for review see (Wilke et al., 2007)). The molecular composition together with the secreted products of the microbiota results in a pH range of the stratum corneum between 4.1 and 5.8 (Proksch, 2018). Acidification of the skin’s surface is critical to maintaining a healthy skin environment, as antimicrobial peptides, such as dermcidin, require an acidic pH for their action (Malik et al., 2016). In inflammatory diseases, such as atopic dermatitis (AD), the skin exhibits an elevated pH value, which contributes to the inability to form a healthy skin environment (Panther and Jacob, 2015).

### Bacterial adaptation to the skin environment

Healthy human skin is rarely colonized with *S. aureus* (Shi et al., 2016), in contrast to the skin of AD patients (Schlievert et al., 2010). Therefore, to date, there are no gene expression analyses of *S. aureus* colonizing healthy human skin. However, some insights were gained using skin explant models (Burian et al., 2021; Cruz et al., 2021). For *S. epidermidis* expression data from healthy skin are available (Teichmann et al., 2022).

Using human skin explants cultivated at the air-liquid interface for up to 8 days, we could mimic skin colonization of *S. aureus*. Similar to the expression profile in the human nose, we provided evidence for significant downregulation of the global virulence regulator *agr* and its target genes *hla* and *psm* during co-culture (Burian et al., 2021) (**Figure 1**). In contrast, the alternative sigma factor B (*sig*B) and its target genes (*clf*A and *fnb*A) as well as the antimicrobial peptide-sensing system (*gra*RS) were strongly upregulated upon skin contact. At later time points, transcription of molecules involved in immune evasion (*scn* and *sak*) and WTA synthesis (*tag*O) was induced. Similar to the expression profile in the nose, enzymes involved in cell wall metabolism (*sce*D and *atl*A) were highly transcribed during co-culture. Interestingly, proteases from all three catalytic classes were strongly induced during the entire colonization process (Burian et al., 2021).

Gene expression was also analyzed using a similar human skin explant model in which the *stratum corneum* was “tape-stripped” to mimic barrier dysfunction. This procedure allowed invasion of *S. aureus* from the epidermis to the dermis (Cruz et al., 2021). Similar to analysis of un-disturbed skin explants (Burian et al., 2021) *asp*23, *clf*A, *atl*A and the protease genes (*aur, ssp*A, *ssp*B) were upregulated upon skin contact (**Figure 1**). Additionally, genes encoding part of the ESAT-6 secretion system (*esx*A, *esx*B, *esx*C, *esa*A, and *ess*B), immunodominant antigens (*isaA* and *isaB*), conserved staphylococcal antigens (*csa*1A and *csa*2) and adhesion proteins (*ebpS* and *sasF*) were found activated after skin inoculation. The highly similar gene expression pattern observed in both skin explant studies indicate that deeper invasion of the strains does not per se induce major changes in gene expression. Only, *hla* promoter activity was shown to be enhanced inside the sweat glands and ducts but not on the skin surface (Cruz et al., 2021).

Recently*, S. epidermidis* gene expression from skin and nose specimens from the same patients were compared (Teichmann et al., 2022). Gene expression was mostly congruent between both sides and characterized by strong induction of adhesion and immune evasion genes (*sdr*G, *cap*C, *dlt*A and *sce*D), as well as *sph* and a putative chitinase (SE0760) (**Figure 1**). However, *agr* activity was low in the nose but readily present on the skin. A similar expression profile was also identified for SE0760, whereas *sceD* and the wall teichoic acid (WTA) biosynthesis gene *tagB* were more pronounced in the nose specimens.

## Conclusion and outlook

The still limited data on gene expression during colonization of the nose or healthy skin indicate that the bacteria are actively growing, adapted to adhere to the underlying tissue and are kept in a non-toxic state by down-regulation of major virulence regulators and their target genes. However, one may assume that gene expression drastically changes once the bacteria enter deeper tissues or the blood stream. Pulia and colleagues demonstrated that gene expression was significantly different when comparing pus samples and wound swabs (Pulia et al., 2022). For example, a relative increase in the expression of toxin genes and virulence regulators (*agr* and *sae*) was observed in purulent material (Pulia et al., 2022). Higher strain toxicity is also indicated by analyses of human cutaneous abscesses (Loughman et al., 2009; Date et al., 2014). Quorum and/or defensin sensing may be major triggers for the switch towards higher toxicity. One can also assume that colonization of non-healthy skin impact gene expression. Recently, Poh and colleagues analyzed the expression of *S. aureus* virulence factors on lesional and non-lesional skin of AD patients. Of the genes investigated, *scn* (encoding staphylococcal complement inhibitor) and the protein A-encoding gene *spa* were the two most highly expressed genes in atopic skin (Poh et al., 2022).

Each habitat is characterized by specific conditions (pH, temperature, nutrient availability, and microbiota) and the habitat can even be subdivided into microenvironments. Thus, gene expression is controlled by a variety of different stimuli. Various tools (metabolomics, improved cell culture techniques, global transcriptome analyses) have been developed to tackle this issue. However, we are still far from knowing the major triggers acting *in vivo* and which regulatory circuits and i-modulons determine specific niche adaptation. Controlled switches likely determine the severity and/or chronicity of infections. More comprehensive gene analyses, sophisticated imaging and metabolomics from different infection sites are required to understand the transition from commensal to pathogenic lifestyles. Such insight can guide new anti-infective strategies to suppress bacterial growth and virulence.

## Author contributions

MB, CW and AY contributed to the manuscript writing. All authors contributed to the article and approved the submitted version.

## Funding

This work was supported by a grant from the START-Program of the Faculty of Medicine of the RWTH Aachen University and by the Deutsche Forschungsgemeinschaft (TRR 156, 246807620 and FOR2497, 289113135 to AY and SPP2225, 446507619 to CW and infrastructural funding of the Cluster of Excellence EXC 2124 “Controlling Microbes to Fight Infections).

## Conflict of interest

The authors declare that the research was conducted in the absence of any commercial or financial relationships that could be construed as a potential conflict of interest.

## Publisher’s note

All claims expressed in this article are solely those of the authors and do not necessarily represent those of their affiliated organizations, or those of the publisher, the editors and the reviewers. Any product that may be evaluated in this article, or claim that may be made by its manufacturer, is not guaranteed or endorsed by the publisher.
